# HIV-1 Subtypes and Recombinants in Northern Tanzania: Distribution of Viral Quasispecies

**DOI:** 10.1371/journal.pone.0047605

**Published:** 2012-10-31

**Authors:** Ireen E. Kiwelu, Vladimir Novitsky, Lauren Margolin, Jeannie Baca, Rachel Manongi, Noel Sam, John Shao, Mary F. McLane, Saidi H. Kapiga, M. Essex

**Affiliations:** 1 Kilimanjaro Christian Medical Centre and College, Tumaini University, Moshi, Tanzania; 2 Kilimanjaro Reproductive Health Program, Moshi, Tanzania; 3 Department of Immunology and Infectious Diseases, Harvard School of Public Health, Boston, Massachusetts, United States of America; 4 London School of Hygiene and Tropical Medicine, London, United Kingdom; St. James School of Medicine, Anguilla

## Abstract

This study analyzed the distribution and prevalence of HIV-1 subtypes, multiplicity of HIV-1 infection, and frequency of inter-subtype recombination among HIV-1-infected female bar and hotel workers in Moshi, Kilimanjaro Region, Tanzania, from 2004 to 2007. The HIV-1 viral sequences spanning the V1-C5 region of HIV-1 *env* gp120 were analyzed from 50 subjects by single genome amplification and sequencing (SGA/S) technique. A total of 1740 sequences were amplified and sequenced from the HIV-1 proviral DNA template. The median *env* sequences analyzed per subject per two time points was 38 (IQR 28–50) over one year of HIV infection. In a subset of 14 subjects, a total of 239 sequences were obtained from HIV-1 RNA template at the baseline visit. The most prevalent HIV-1 subtypes were A1 (56%) and C (30%), while HIV-1 subtype D and inter-subtype recombinant viruses were found in 6% and 8% of subjects respectively. Transmission of multiple HIV-1 variants was evident in 27% of the subjects infected with pure HIV-1 subtypes A1, C, or D. The HIV-1 inter-subtype recombinants were found in 8% including HIV-1 C/A, D/A, and complex mosaic recombinants. Multiple viral variants were found in two subjects infected with inter-subtype recombinants. One subject harbored quasispecies of both pure HIV-1 A1 and C/A recombinant. The other subject was infected with two complex mosaic inter-subtype recombinant variants belonging to subtype D. HIV-1 multiple infections and ongoing recombination contribute significantly to the genetic diversity of circulating HIV-1 in Tanzania and have important implications for vaccine design and the development of therapeutic strategies.

## Introduction

Based on phylogenetic inference, human immunodeficiency virus type 1 (HIV-1) can be classified into four groups: main group (M), outlier group (O), non-M-non-O group [1] and group P [2], [3]. Group M accounts for the majority of HIV-1 infections globally and is divided into nine genetic subtypes (A, B, C, D, F, G, H, J, and K). However, HIV-1 subtypes A and C are the most prevalent in the worldwide epidemic [4], [5]. HIV-1 subtypes A and F are further subdivided into sub-subtypes A1–A4 and F1–F2, respectively [6]. Viruses from different lineages within group M recombine and form recombinant viruses [1], [7]. Currently, 51 circulating recombinant forms (CRFs) and a large number of unique recombinant forms (URFs) have been described (see http://www.hiv.lanl.gov), providing evidence for ongoing evolution of HIV-1 in the global epidemic [8].

Furthermore, individuals infected with multiple HIV-1 subtypes [9]–[18], as well as individuals infected with multiple strains of the same HIV-1 subtype have been reported [19]–[23], indicating that the immune response generated against one strain or subtype of HIV-1 does not necessarily protect against other HIV-1 strains or subtypes [24]–[26].

HIV-1 infections with multiple viral variants and recombinant viruses present a challenge for vaccine development and clinical management. Multiple HIV-1 infections and recombinant viruses have been associated with increased disease progression [27]–[29]. Individuals infected with a drug-sensitive virus may be super-infected with a drug-resistant strain, severely hindering antiviral treatment options [30].

Tanzania is one of the countries in sub-Saharan Africa that has been most severely affected by the HIV epidemic. Several molecular epidemiological studies have reported that HIV-1 subtypes A1, A2, C, D, G, CRF10_CD and other inter-subtype recombinant viruses co-circulate in Tanzania [31]–[43]. Studies in cohorts of female bar and hotel workers, a high-risk population of HIV-1 infection in Moshi town, Kilimanjaro region, and in the Mbeya region, reported HIV-1 prevalence rates of 26.3% [44] and 67.8% [10], respectively, indicating that HIV-1 infection was a major public health problem in this population. HIV-1 subtypes A1, A2, C, D, CRF10_CD and other inter-subtype recombinant viruses were reported among bar and hotel workers in Moshi town [34], [36]. The HIV-1 subtype profile was similar in the Mbeya region with the exception of subtype A2 and CRF10_CD which have not been found in the region [10]. Furthermore, HIV-1 multiple variants were reported in 23% of a high-risk population of female bar and hotel workers with acute HIV-1 infection in the Mbeya region of Tanzania [16].

It is likely that female commercial sex workers and their male clients, as well as female bar and hotel workers, contribute substantially to the rapid expansion of the HIV-1 epidemic in developing countries [45], [46] and may play an important role in evolution facilitating multiple infections and recombination events [10].

There is little information about multiplicity of HIV-1 infections in regions like Tanzania where HIV-1 multiple subtypes and inter-subtype recombinant viruses co-circulate.

This study focused on the molecular epidemiology of HIV-1 epidemic in female bar and hotel workers in Moshi town, Kilimanjaro region, Tanzania. We determined the distribution and prevalence of HIV-1 subtypes, multiplicity of HIV-1 infection, and frequency of inter-subtype recombinant viruses in Tanzania. We analyzed samples from a prospective cohort study of women at increased risk of HIV-1 infection in Moshi town, Kilimanjaro region of Tanzania. The objective of the study was to determine factors associated with HIV-1 infection and describe the molecular epidemiology of the HIV-1 epidemic over the course of the study.

## Materials and Methods

### Ethics Statement

This study was conducted according to the principles expressed in the Declaration of Helsinki, and was approved by the institutional review boards of the Kilimanjaro Christian Medical Centre (KCMC), the Tanzania National Institute for Medical Research, and the Harvard School of Public Health (HSPH). All study subjects provided written informed consent for the collection of samples and subsequent analysis.

### Study subjects

Between December 2004 and March 2007, we recruited and followed-up 800 women employed in the hotels, bars and guest houses located in the study area. The age of the subjects at enrollment ranged from 16 to 55 years old. All enrolled subjects were females. Following baseline assessment, each subject was evaluated quarterly for a period of one year. At each visit subjects were examined and interviewed to obtain information about sexual behaviors and HIV-related risk factors.

The HIV-1 infections were identified by using two diagnostic HIV enzyme-linked immunosorbent assays (ELISA), Enzygnost Anti HIV1/2 Plus (Dade Behring Liederbach, Germany) and Vironostika HIV-Uni-form II plus (Organon, Boxtel, Netherlands). Indeterminate or discordant results were resolved by Western blot test (Bio-Rad Laboratories Ltd, Dartford, UK).

Among 800 subjects, 139 (17%) were HIV-1 positive by serological testing. Blood and genital samples were collected from HIV-1 positive subjects at each visit. For this study, a subset of 50 subjects with samples available at baseline and 12 months were selected. The mean age of selected subjects was 31.7 years old, ranging from 21 to 53 years. The raw data for age, HIV-1 plasma viral load, number of HIV-1 viral and proviral DNA sequences, pairwise mean genetic distances and multiplicity of HIV-1 infection are shown in Table S1.

### Single-genome amplification and sequencing (SGA/S)

Peripheral blood mononuclear cells (PBMCs) were isolated from the whole blood by the ficoll-hypaque density gradient centrifugation method, and stored in the liquid nitrogen. HIV-1 genomic DNA was extracted from PBMCs by QIAamp DNA Blood Mini Kit (QIAGEN) according to the manufacturer's recommendations.

A fragment of HIV-1 *env* gp 120 spanning the V1–C5 region (nucleotide position 6,615–7,757; HXB2 numbering; see http://www.hiv.lanl.gov) was amplified using modified SGA/S technique [47], [48] based on the limiting dilutions method [49]. The template was serially diluted and PCR amplified at each dilution. The template dilution that produced approximately 30% of positive amplicons was considered to be a dilution with a single amplifiable molecule. Two time points, baseline and 12 months were targeted in this study. The median *env* DNA sequences analyzed per subject per two time points was 38 (IQR 28–50). First-round reaction was conducted using primer pairs ED3 and ED14 [50]. PCR reactions were performed in a volume of 20 µl and contained 1 µl of proviral DNA, 1.8 mM FastStart High Fidelity Buffer (Roche), 10 mM deoxynucleotide triphosphate (dNTPs (dATP, dCTP, dGTP and dTTP)) Roche)), 10 pmol of each primer (Integrated DNA Technologies) and 5U FastStart High Fidelity Enzyme (Roche). The second round PCR reaction was done with primer pairs ED5 and ED12 [50] 3 µl of first-round PCR product diluted 1∶50 and 22 µl of master mix containing 1.8 mM FastStart High Fidelity buffer, 10 mM dNTPs, 10 pmol of each primer and 5 U FastStart High Fidelity Enzyme. Thermal cycling conditions for both reactions were: 95°C for 2 minutes, followed by 3cycles at 95°C for 1 min, 57°C for 1 min, and 72°C for 2 min followed up by 37 cycles of 95°C for 15 sec, 57°C for 45 sec and 72°C for 1 min, with a final extension step at 72°C for 7 min. Reaction mixtures were stored at 4°C until use.

### RNA isolation, reverse transcriptase polymerase chain reaction (RT-PCR) and SGA/S

We identified 14 out of 50 subjects infected with HIV-1 multiple variants. To exclude potential laboratory contamination, the alternative amplification template, the viral RNA from plasma, was used in a subset of 14 subjects. HIV-1 viral RNA was isolated from plasma sample by QIAamp viral RNA Mini Kit (QIAGEN, Valencia, CA) according to the manufacturer's recommendations. The cDNA synthesis was performed using the Transcriptor First Strand cDNA Synthesis Kit (Roche Diagnostics GmbH, Mannhein, Germany). Viral RNA, 10 pmol of OFM19 primer (nucleotides 9,604 to 9,632; HXB2 numbering) and nuclease-free water were incubated at 65°C for 10 min to denature the secondary structure of the RNA. The cDNA synthesis was performed in a volume of 20 µl containing denatured viral RNA, 8 mM reverse transcriptase buffer, 20 U RNase Inhibitor, 10 mM dNTPs, and 10 U transcriptor reverse transcriptase enzyme. The reverse transcriptase reaction was incubated at 55°C for 30 min followed by heating at 85°C for 5 min to inactivate the enzyme. The cDNA samples were stored at −80°C until use.

The cDNA synthesized from the viral RNA template was amplified by the SGA/S technique similar to PCR amplification of proviral DNA. First-round PCR reactions were conducted using primer pairs OFM19 and VIF1 (5′-GGG TTT ATT ACA GGG ACA GCA GAG-3′) (nucleotides 4,900 to 4,923; HXB2 numbering). PCR reactions were performed in a volume of 22 µl containing 1 µl of cDNA, 1.8 mM FastStart High Fidelity buffer, 10 mM dNTPs, 10 pmol of each primer and 5 U FastStart High Fidelity Enzyme. The first PCR condition consisted of 95°C for 2 minutes, followed by 35 cycles of 95°C for 15 sec, 57°C for 30 sec, 68°C for 4 min and extension step 68°C. The second round PCR reaction was done with primer pairs ED5 and ED12. Three µl of first-round PCR product diluted 1∶20 were added to 22 µl of master mix containing 1.8 mM FastStart High Fidelity Buffer, 10 mM dNTPs, 10 pmol of each primer and 5 U FastStart High Fidelity Enzyme. The second round PCR was performed at 95°C for 2 min, followed by 35 cycles of 95°C for 20 sec, 60°C for 30 sec, 72°C for 2 min, followed by final extension step at 72°C for 7 min. Reaction mixtures were stored at 4°C until use.

Amplified products from both proviral DNA and viral RNA were electrophoretically analyzed by applying 5 µl of second PCR amplification product to 1% agarose gel containing ethidium bromide, and visualized under ultraviolet light. Amplicons were purified by Exo-Sap [51] and directly sequenced on both strands on the ABI 3730 DNA analyzer using the BigDye technology.

### Phylogenetic analysis

Generated HIV-1 proviral DNA and viral RNA sequences of the gp 120 *env* V1–C5 region were assembled and edited using SeqScape V 2.6. The sequences were aligned together with the HIV-1 reference subtype sequences retrieved from the Los Alamos HIV-1 Sequence Database at http://www.hiv.lanl.gov using muscle algorithm in MEGA 5.0 [52] with minor manual adjustment in BioEdit version 7.0 [53]. Maximum likelihood (ML) phylogenetic trees were constructed by PhyML version 3.0.1 [54] and visualized by FigTree v1.3.1 [55]. The approximate likelihood ratio test (aLRT) was used as a statistical test for support of splits [56]. aLRT values ≥0.95 were considered significant and are displayed at the tree nodes. Additionally, the neighbor-joining (NJ) trees were constructed by MEGA 5.0 using the Kimura-two parameter model for pairwise distance calculations with 1000 bootsrap replicates [57]. The bootstrap values ≥80% were considered significant [58]. HIV-1 subtypes were determined based on branching topology, clustering and splits support of the analyzed sequences and their phylogenetic relationships with HIV-1 reference subtype sequences from the Los Alamos HIV-1 Sequence Database.

The DNA sequences were analyzed for evidence of APOBEG3G induced hypermutation by using Hypermut tool V2.0 [59]. Sequences with a P value of ≤0.05 were considered enriched for mutations consistent with APOBEG3G signatures and were excluded from analysis.

### Multiplicity of HIV-1 infection

In order to distinguish HIV-1 infections with single and multiple viral variants, phylogenetic analysis was performed within the subset of subjects with HIV-1 subtype A1 (n = 27), subtype C (n = 15) and subtype D (n = 3). Four subjects were infected with inter-subtype recombinant viruses and therefore excluded from this analysis. (Note that 28 subjects were infected with subtype A1; however, one (code 181) out of 28 subjects had one DNA sequence and was excluded in this analysis).

Decision on multiplicity of HIV-1 infection was made based on branching topology and clustering of analyzed viral quasispecies. The HIV-1 single variant was defined as an infection of the same HIV-1 subtype in which the viral quasispecies form a distinct phylogenetic cluster with an aLRT support value ≥0.95 in ML analysis, and/or a bootstrap value ≥80% in NJ analysis. The HIV-1 multiple variant was defined as an infection resulting from more than one variant of the same or different HIV-1 subtypes in which the viral quasispecies formed more than one distinct cluster each supported with an aLRT support value ≥0.95 in ML analysis, and/or a bootstrap value ≥80% in NJ analysis. Mean pairwise genetic distances between the clusters were computed for subjects infected with multiple HIV-1 variants.

Nucleotide differences were visualized by the highlighter tool from the Los Alamos HIV-1 Sequence Database website by comparing the HIV-1 proviral DNA and viral RNA sequences to the consensus of the earliest sequences created in BioEdit.

### Screening for inter-subtype recombination and breakpoints identification

All DNA sequences (n = 1979) generated in this study were screened for evidence of inter-subtype recombination by the Recombination Identification Program (RIP 3.0) [60] and the REGA HIV-1 Subtyping Tool-Version 2.0 [61]. The identified recombinant viruses were further analyzed for subtype assignment and breakpoints identification by SimPlot software v3.5.1 [62]. The bootscanning method in SimPlot was parameterized with sliding window size of 350 bp, step increment 3 bp, and Kimura 2-parameter distance model for inferring phylogenetic trees by the NJ method. HIV-1 reference subtype sequences retrieved from the Los Alamos HIV Sequence Database were used. Identified breakpoints were visually inspected in BioEdit. To confirm the HIV-1 subtypes in the inter-subtype recombinant viruses, nucleotide sequences on both sides of the breakpoint were analyzed independently by re-constructing phylogenetic trees using the splits at the putatively identified breakpoints.

### HIV-1 RNA load from Plasma

For each subject, the HIV-1 plasma RNA viral load was quantified by the Roche Amplicor HIV-1 Monitor test, version 1.5 assay as recommended by manufacturer (Roche Molecular Systerms) at the baseline and the 12 month visits.

### Viral diversity

Mean pairwise genetic distances were computed between baseline and 12 month visits using MEGA software V 5.0. Multiple viral quasispecies were available at two visits in 24 of 27 subjects with subtype A1, and 14 of 15 subjects with HIV-1 subtype C. For subtype D and inter-subtype recombinants the mean pairwise genetic distances were calculated in three and four subjects, respectively.

### Control for cross-contamination

To confirm that the HIV-1 multiple variants reported in this study were not due to laboratory cross-contamination during specimen collection, processing, amplification, and/or sequencing, the following strategies were practiced consistently during the experimental work: (1) viral RNA plasma was used as an alternative template (n = 14); (2) sample from an alternative study visit was amplified and sequenced; (3) nucleic acid isolation and different steps of PCR procedures were separated by place and time; and (4) proper quality controls were included at each step.

### Statistical analysis

Descriptive statistics were quantified using Sigma Stat v.3.5. Comparisons of continuous outcomes between two groups were based on the Mann-Whitney Rank Sum test. The bootstrap and aLTR support values for splits in the inferred phylogenetic trees were computed by MEGA 5.0 and PhyML respectively. All reported p-values are 2-sided and not adjusted for multiple comparisons.

### Accession numbers

All generated sequences were submitted to GenBank. The accession numbers are JX070938–JX072916.

## Results

### Phylogenetic relationship and subtype distribution

A fragment of HIV-1 genome encompassing the V1-C5 region of *env* gp120 was analyzed by SGA/S technique in 50 subjects. A total of 1,740 viral sequences were amplified and sequenced from proviral DNA. The median number of *env* sequences analyzed per subject per two time points was 38 (IQR 28–50). In a subset of 14 subjects, a total of 239 sequences were obtained from HIV-1 RNA template at the baseline visit. In four subjects (codes 181, 321, 404, and 405) samples could only be amplified at the 12 month visit, while in one subject (code 740) only the baseline sample was successfully amplified (Table S1).

The HIV-1 subtyping of viral sequences generated in this study was performed by alignment with HIV-1 reference subtypes (A, B, C, D, F, G, H, J and K) from the Los Alamos HIV-1 Sequence Database, and phylogenetic analysis by Maximum Likelihood (ML) method implemented in FastTree 2 [63]. Viral sequences from 28 subjects clustered with HIV-1 subtype A1 references (aLRT support of 1.0), viral sequences from 15 subjects clustered with HIV-1 subtype C references (aLRT support of 1.0), and viral sequences from three subjects clustered with HIV-1 subtype D references (aLRT support of 1.0) (data not shown). 183 HIV-1 DNA recombinant sequences (inter-subtype) from four subjects were analyzed separately (see below).

**Figure 1:**
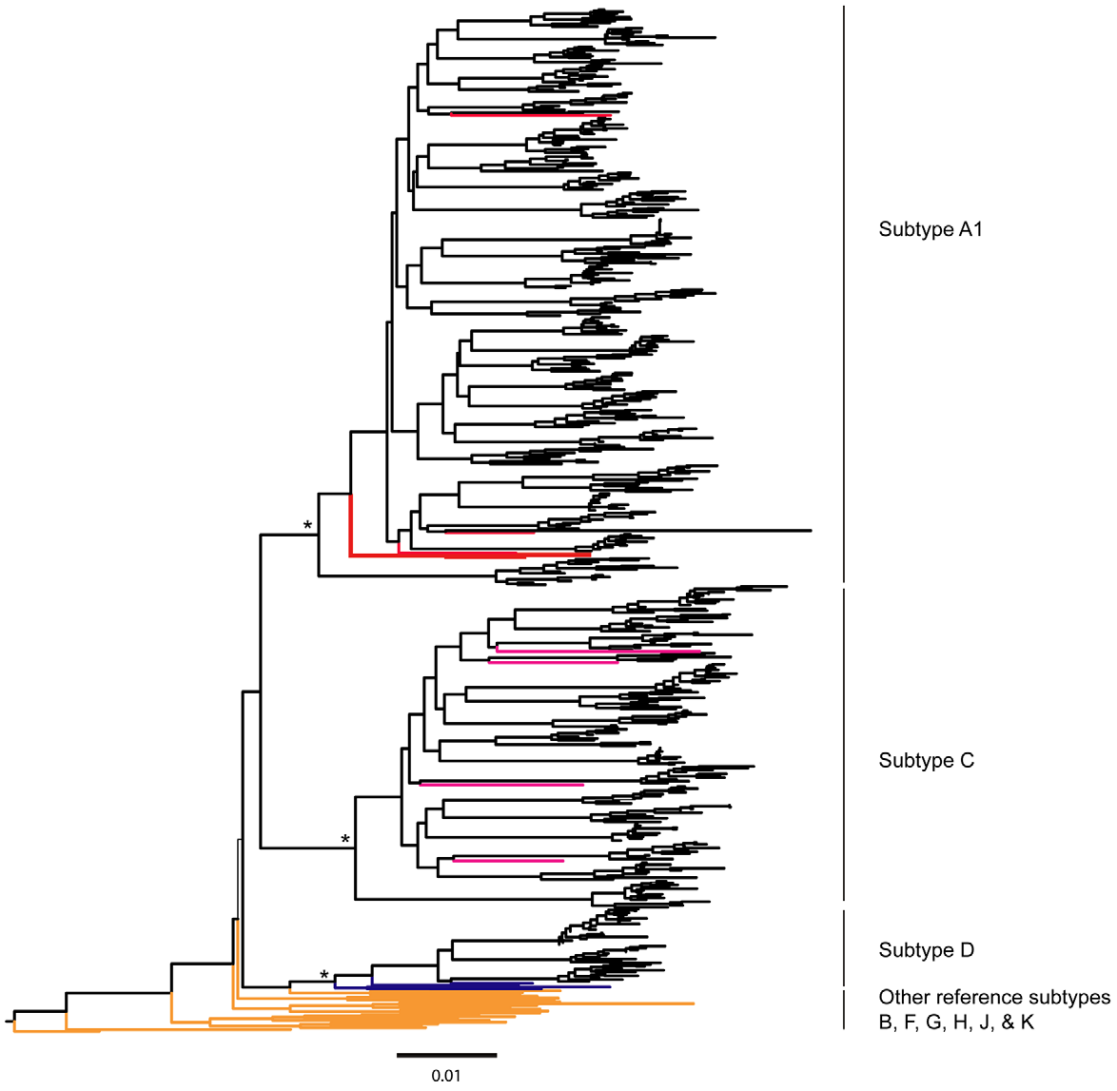
A maximum likelihood (ML) phylogenetic tree of HIV-1 env gp 120 V1-C5 sequences from 50 subjects. The ML tree was constructed by PhyML 3.0.1 and visualized in FigTree. HIV-1 group N sequence was used as an outgroup. A representative subset of 516 out of 1796 DNA sequences generated in this study is presented in the Figure. Ten DNA sequences representative of each sample were chosen and analyzed with HIV-1 reference subtypes from the Los Alamos HIV Sequence Database. The inter-subtype recombinant DNA sequences were excluded in this Figure. Reference subtypes A1, C, D are shown in red, pink, and blue respectively. The other reference subtypes are shown in orange. The approximate likelihood ratio test (aLRT) values of ≥0.95 were considered significant and are shown by an asterisk (*). Scale at the bottom of the Figure corresponds to 0.01 nucleotide substitutions per site.

For clarity of presentation, ten proviral DNA sequences per subject were selected and analyzed for HIV-1 subtyping. This subset included the first five sequences from the baseline visit and the first five sequences from the 12 month visit. For the subjects with less than five sequences, all available sequences were included (n = 7). If viral sequences were available at one time point (n = 5) only, ten sequences were selected and included in the analysis. In addition, from 14 subjects with viral sequences obtained from HIV-1 RNA template at the baseline visit, the first five sequences were selected and included in the HIV-1 subtyping analysis. The phylogenetic inference using the ML method (Fig. 1) revealed that the most prevalent HIV-1 subtype was A1 (56%), followed by subtype C (30%). HIV-1 subtype D and inter-subtype recombinant viruses were less prevalent (6% and 8% respectively). The distribution of HIV-1 subtypes among study subjects is summarized in Table 1.

**Table 1:** Distribution of HIV-1 *env V1-C5* subtypes among female bar and hotel workers in Moshi, Kilimanjaro region, Tanzania, during 2004–2007.

HIV-1 subtype	Study subjects, (%)
A1	28 (56%)
C	15 (30%)
D	3 (6%)
Inter-subtype recombinants	4 (8%)
Total	50 (100%)

### Multiplicity of HIV-1 infection

**Figure 2:**
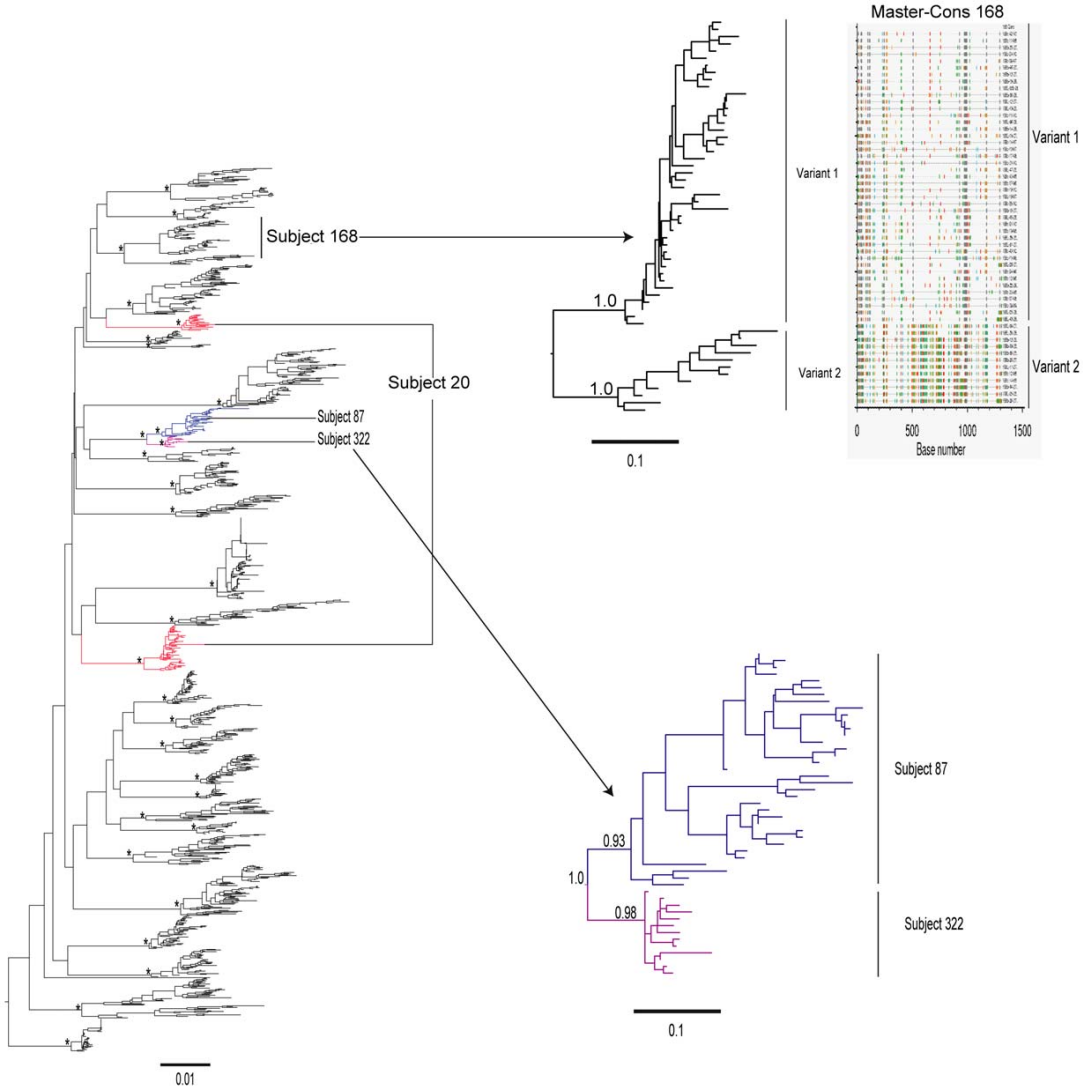
A maximum likelihood phylogenetic tree of HIV-1 env gp 120 V1-C5 sequences from 29 subjects infected with HIV-1 subtype A is presented. (Note that 13 DNA sequences classified as pure subtype A1 from subject 322 were also included in this analysis, resulting in a total of 29 subjects). Subject 20 was infected with two distinct unrelated variants of HIV-1 subtype A1 which are shown in red. Subject 168 infected with two closely related variants of subtype A1 which are shown in light blue and highlighter plot. The DNA sequences of subject 87 shown in blue clustered with the DNA sequences of subject 322 shown in purple with the aLRT support value of 1.0. Methodological details have been described in the legend to Figure 1. Scale at the bottom of the Figure corresponds to 0.01 nucleotide substitutions per site.

In order to determine HIV-1 infection with multiple variants of the same subtype, phylogenetic analysis was performed within the subsets of individuals infected with HIV-1 subtype A1 (n = 27) and subtype C (n = 15). The inferred ML phylogenetic trees helped to determine that single HIV-1 variants were found in 33 (73.3%) subjects (an example is shown in Fig. S1), while multiple HIV-1 variants were evident in 12 (26.7%) subjects. In HIV-1 subtype A1, nine of 27 (33%) subjects had HIV-1 multiple variants (Table 2). Two distinct phylogenetic lineages (mean pairwise distance between clusters of 18%; aLRT support 1.0, Fig. 2) were observed for subject 20, suggesting infection with two unrelated variants of HIV-1 subtype A1. Similarly, two distinct clusters (mean pairwise distance between clusters 19.6%) were observed for subject 87, suggesting that this subject was also infected with two unrelated variants of HIV-1 subtype A1 (data not shown). The DNA sequences of the other seven subjects formed multiple distinct clusters with significant aLRT support values and mean pairwise distances from 6.4% to 12.4%, indicating uncertainty in relation to transmission of unrelated, or closely related viral variants. An example of infection with two closely related variants of HIV-1 subtype A1 is shown in Fig. 2.

**Table 2:** HIV-1 single and multiple variants of the same subtype among female bar and hotel workers in Moshi, Kilimanjaro region, Tanzania.

HIV-1 subtypes	Subjects, n	HIV-1 infection with single variant	HIV-1 infection with multiple variants
A1	27	18 (67%)	9 (33%)
C	15	12 (80%)	3 (20%)
D	3	3 (100%)	0 (0.0%)
Total	45	33 (73.3%)	12 (26.7%)

* A subset of 45 samples with pure subtypes (A1, C or D) was analyzed for multiple infections; sample 181 was excluded due to a single available DNA sequence.

**Figure 3:**
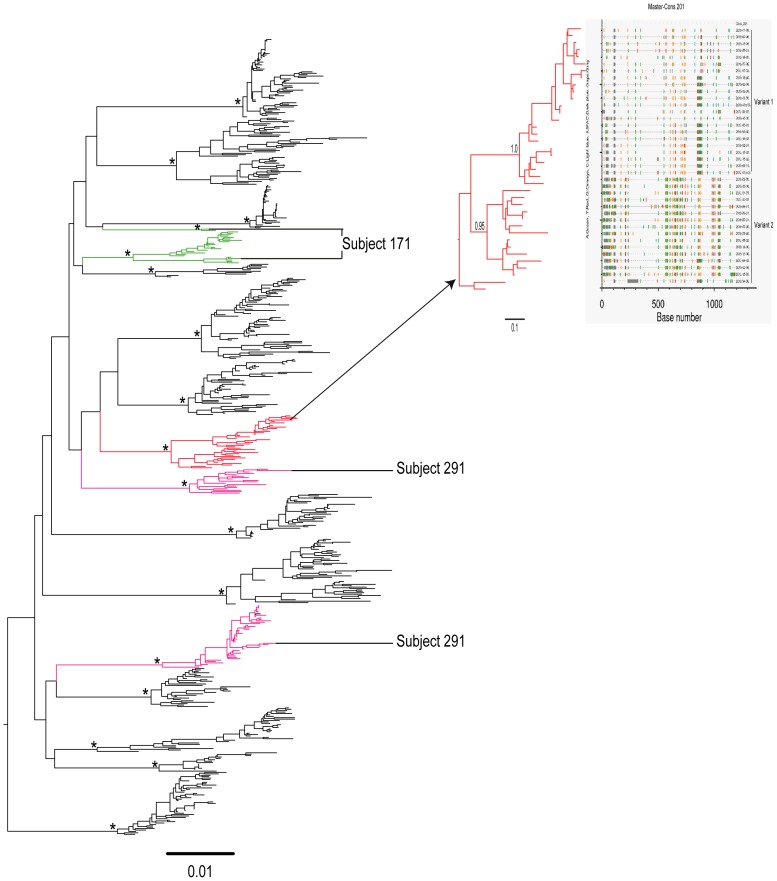
A maximum likelihood phylogenetic tree of HIV-1 env gp 120 V1-C5 sequences from 15 subjects infected with subtype C. Subjects 171 and 291 were infected with two unrelated HIV-1 variants of subtype C which are shown in green and pink respectively. Subject 201 infected with two closely related variants of subtype C which are shown in red and highlighter plot. Methodological details have been described in the legend to Figure 1. Scale at the bottom of the Figure corresponds to 0.01 nucleotide substitutions per site.

Phylogenetic analysis of HIV-1 subtype C sequences from 15 subjects indicated that three subjects (20%) had HIV-1 multiple variants. Viral sequences in subjects 171 and 291 formed two distinct phylogenetic lineages with mean pairwise distances between two clusters of 16.3% and 20.6%, respectively, and a high aLRT support value (Fig. 3), indicating multiple HIV-1 subtype C infections. The viral sequences from subject 201 formed two distinct clusters, each with a significant aLRT support value, but the mean pairwise distance between two clusters was 11.4%, indicating uncertainty in relation to transmission of unrelated, or closely related, HIV-1 subtype C variants. An example of infection with closely related HIV-1 subtype C variants is shown in Fig. 3. None of the subjects with pure HIV-1 subtype D were infected with multiple variants (Table 2).

### A potential transmission cluster

Within the analyzed set of viral sequences we found a potential transmission cluster evident from branching topology and aLRT support of quasispecies belonging to subjects 322 and 87 (aLRT support 1.0; Fig. 2). This observation indicates a potential epidemiological link due to a common source of infection or existence of a larger HIV-1 transmission cluster. To confirm that the clustering was not due to laboratory cross-contamination, the cDNA sequences from the baseline visit of subject 322 were also included in the analysis. Results showed that four cDNA sequences and nine DNA sequences from subject 322 clustered together with 36 sequences from subject 87. Taken together, these results suggest no laboratory cross-contamination.

### Inter-subtype recombinant viruses

All viral DNA sequences generated in this study were analyzed for evidence of inter-subtype recombination by the recombination identification program (RIP 3.0; http://www.hiv.lanl.gov), and REGA HIV-1 subtyping tool (http://dbpartners.stanford.edu/RegaSubtyping/). The inter- subtype recombinant viruses were confirmed by bootscanning analysis using SimPlot v3.5.1 [62].

HIV-1 inter-subtype recombinant viruses were found and analyzed in greater detail in four (8%) out of 50 subjects (Table 3). In three subjects, 33, 471, and 510, all viral quasispecies were represented by inter-subtype recombinant viruses, while subject 322 had both pure HIV-1 subtype A1 and inter-subtype recombinant viruses. All 48 sequences of subject 33 were classified as inter-subtype recombinant viruses of HIV-1 subtype D/A with a similar breakpoint at position 7,483 (HXB2 numbering). HIV-1 viral sequences of subject 471 (n = 50) were classified as C/A1 inter-subtype recombinant viruses with a similar breakpoint at position 7,085 (HXB2 numbering). In subject 322, 13 of 41 viral quasispecies were pure HIV-1 subtype A, while the remaining 28 sequences were represented by HIV-1 C/A recombinant with a breakpoint located at position 6,881 (HXB2 numbering). To determine relationships between the non-recombinant subtype A and the A fragment in C/A recombinant sequences, the sub-region at position 6,881–7,745 (HXB2 numbering) was phylogenetically analyzed. We found that 13 non-recombinant HIV-1 A sequences clustered separately from the subtype A1 fragment from HIV-1 C/A recombinant virus (data not shown).

**Table 3:** Inter-subtype recombinant viruses distribution among female bar and hotel workers in Moshi, Kilimanjaro region, Tanzania, during 2004–2007.

Subject's ID	Proviral DNA sequences, n	Viral cDNA sequences, n	HIV-1 inter-subtype recombinants	SimPlot bootscan breakpoint position*
33	47	1	D/A	7,483
322	9	4	A	
	23	5	C/A	6,881
471	50	NA	C/A	7,085
510	47	9	D/U§	7,226
	1	NA	D/U§/D	7,346: 7,500

§ Unclassified region.

NA: not available.

* Numbering positions relative to HIV HXB2.

**Figure 4:**
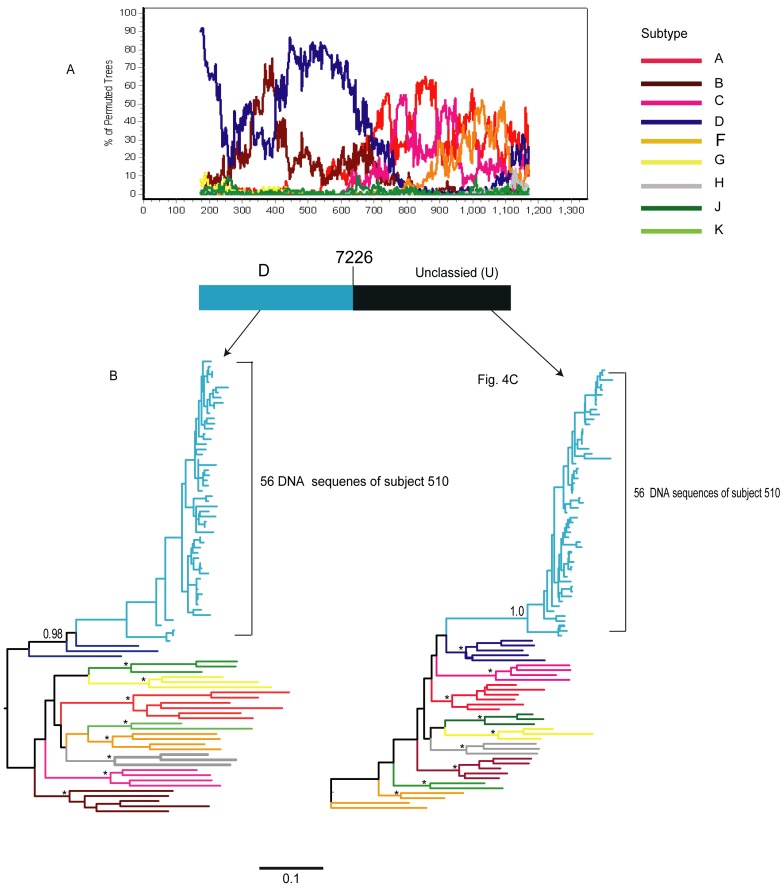
Maximum likelihood phylogenetic trees for two fragments of the 56 out of 57 viral quasispecies for subject 510 as identified by SimPlot analysis. Fig. 4A: In subject 510, the majority (56 of 57) of viral quasispecies were comprised of subtype D (light blue bar) and unclassified regions (back bar). A breakpoint is located at position 7,226 (HXB2 numbering). Bootscanning was performed on the alignment by SimPlot v 3.5.1 using Kimura 2-parameter model with sliding window size 350 bp and step increment 3 bp and Kimura 2-parameter distance model. A bootstrap value ≥80% was considered significant. Bootstrap values are indicated on the y axis and nucleotide positions in the alignment are shown on the x axis. Figs. 4B & 4C: Phylogenetic analysis (ML trees) of two fragments identified by the breakpoint located at position 7,226 (HXB2 numbering). After SimPlot analysis the breakpoint was visualized in the alignment by BioEdit. Fig. 4B is a ML tree of the fragment classified as HIV-1 subtype D and Fig. 4C is a ML tree of the unclassified fragment whose subtype classification was unresolved by bootscan analysis. The unclassified region clustered with none of the HIV-1 reference subtypes from the Los Alamos HIV Sequence Database. The viral quasispecies of subject 510 are shown in light blue, and the legend at the right of the bootscan plot indicates reference HIV-1 subtypes. The aLRT values ≥0.95 were considered significant and are shown by asterisk (*). Selected aLRT values are shown at the branch node of the tree. Scale at the bottom of the Figure corresponds to 0.1 nucleotide substitutions per site.

**Figure 5:**
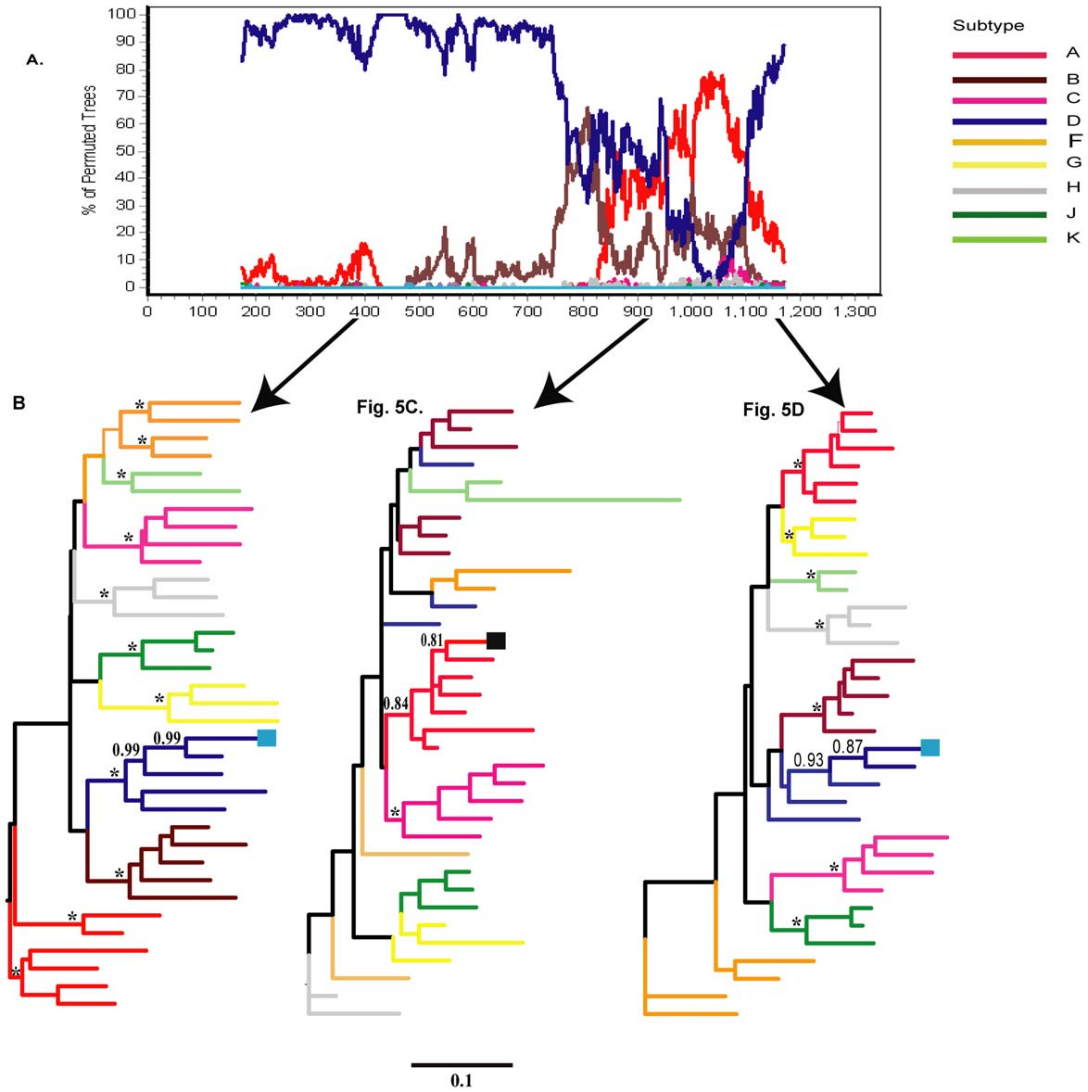
Maximum likelihood phylogenetic trees for three fragments of the one out of 57 viral quasispecies for subject 510 as identified by SimPlot analysis. Fig 5A: A single viral sequence in subject 510 with distinct recombination pattern, D/U/D. The bootscan plot was generated by Simplot. HIV-1 subtype D is shown in the blue bar and unclassified region is shown in the black bar. Figs. 5B, 5C & 5D: Maximum likelihood (ML) trees of three fragments classified as subtype D (nucleotide position 6,615 to 7,345, Fig. 5B), unclassified fragment (nucleotide position 7,346 to 7,500, Fig. 5C) and fragment classified as subtype D (nucleotide position 7,501 to 7,745). The viral quasispecies were analyzed with reference sequences from the Los Alamos HIV Sequence Database. The first fragment clustered with subtype D with a high aLRT support value (shown in light blue square), the second fragment clustered with subtype A1 but aLRT was insufficient (0.81; shown in black square). The third fragment clustered with subtype D with a weak aLRT support (0.87). Scale at the bottom of the Figure corresponds to 0.1 nucleotide substitutions per site.

The SimPlot analysis of viral sequences in subject 510 showed that 56 of 57 sequences were comprised of HIV-1 subtype D and unclassified regions (U) (Fig. 4A). One out of 57 sequences included the subtype A-like insertion on the backbone of HIV-1 subtype D (nucleotides position 7,346 to 7,500; HXB2 numbering), however, the bootstrap support for the insertion was not sufficient for assignment to HIV-1 subtype A1 (Fig. 5A).

To confirm the recombinant patterns of the subset of 56 DNA sequences in subject 510, the ML trees were generated separately for two regions, 6,615 to 7,226 (HXB2 numbering) and 7,227 to 7,745 (HXB2 numbering; Fig. 4B & C). Results showed that the first analyzed fragment clustered with HIV-1 subtype D references (Fig. 4B; aLRT support of 0.98). However, the second fragment did not cluster with any subtype (Fig. 4C), and can be considered as an unclassified region. Similar branching topology and bootstrap support were observed in phylogenetic trees inferred by the neighbor-joining (NJ) method (data not shown).

To clarify the recombination patterns in the DNA sequence of subject 510 that included the subtype A-like insertion on the background of HIV-1 subtype D, the ML phylogenetic trees were inferred for three fragments of this sequence. As expected, the SimPlot results (Fig. 5A) were confirmed by phylogenetic inference (aLRT support of 0.99; Fig. 5B). The second fragment (nucleotides position 7,346 to 7,500; HXB2 numbering) that was close to A1 had an aLRT support of 0.81 which made its assignment with HIV-1 subtype A1 uncertain (Fig. 5C). The third fragment clustered with HIV-1 subtype D with an aLRT support of 0.87 potentially indicating a diversified subtype D variant (Fig. 5D). The low aLRT support value for the second and the third fragments could possibly be due to their short length and limited number of informative sites. Phylogenetic analysis by NJ method showed similar branching topology (data not shown). Therefore, we concluded that subject 510 was infected with two complex mosaic inter-subtype recombinant viruses; one recombinant comprised of subtype D and unclassified regions (U), while the other recombinant included the HIV-1 subtype A-like insertion on the background of HIV-1 subtype D. The subtype A-like fragment was considered to be unclassified region (U).

### HIV-1 RNA load in plasma

The HIV-1 RNA load in plasma was quantified in all subjects at both the baseline and the 12 month visits by the Roche Amplicor HIV-1 Monitor test, version 1.5 assays. Undetectable levels of HIV-1 RNA were found in one subject at the baseline visit and in ten subjects at the 12 month visit (Table S1). Information about anti-retroviral treatment (ART) status was not collected in this study. Therefore it is possible that at least some subjects started ART during the study. The undetectable levels of HIV-1 RNA at 12 month visit support initiation of ART. We addressed whether HIV-1 RNA load in plasma differs between HIV-1 subtypes A, C, D, and inter-subtype recombinant viruses. We found no statistically significant difference in the levels of HIV-1 RNA load between subtypes at two time points (Table S2). High HIV-1 RNA plasma viral load was observed in some of the subjects with multiple HIV-1 subtype A1 infections at both the baseline and the 12 month visits but the difference was not statistically significant (data not shown).

### Viral diversity

Mean pairwise genetic distances within subsets of subjects infected with HIV-1 subtypes A, C, D and inter-subtype recombinant viruses were calculated at two time points over the follow-up period of one year. Changes in mean pairwise distances were not statistically significant over the observed period of time, apparently due to high heterogeneity among subjects and relatively small sample size (Fig. S2). The mean pairwise genetic distance of viral sequences in HIV-1 subtype A1 changed from 3.9±2.3 and 4.6±2.4 (P = 0.26), in HIV-1 subtype C from 5.0±2.9 and 4.9±2.4 (P = 0.88), in HIV-1 subtype D from 2.5±0.5 and 3.4±0.6 (P = 0.09), and in recombinant viruses from 5.3±3.5 and 6.4±3.6 (P = 0.68) (Table S3). 22 (92%) of 24 subjects infected with HIV-1 subtype A1, eleven (79%) of 14 subjects infected with HIV-1subtype C, all three subjects infected with HIV-1 subtype D, and three of four subjects infected with recombinant viruses showed an increase of mean pairwise genetic distances over time. However, there was a significant decrease in the mean pairwise genetic distances of one subject infected with HIV-1 subtype C at the 12 month visit, which may be due to a small number of viral sequences being analyzed (Fig. S2). The overall mean pairwise genetic distances are shown in Table S3. These results indicated that there was no statistical significant difference in mean pairwise genetic distances between the baseline and the 12 month visits for HIV-1 subtypes A1, C, D, and inter-subtype recombinant viruses. We found a wide range of variation of mean viral diversity between subjects from 0.1% to 11.3%.

## Discussion

This study assessed the distribution and prevalence of HIV-1 subtypes, multiplicity of HIV-1 infection, and provided evidence of inter-subtype recombination among female bar and hotel workers, one of the highest risk populations in Tanzania.

The most prevalent HIV-1 subtype was subtype A1 (56%), followed by subtype C (30%), while HIV-1 subtype D (6%) and inter-subtype recombinant viruses (8%) were less frequent. These results are consistent with the previous studies conducted in Tanzania [36], [38] and suggest that HIV-1 subtype A1 still predominates while HIV-1 subtype C continues to spread in this population.

Previous studies from Tanzania, using small sample sizes, reported a predominance of HIV-1 subtype D in the mid-1990s [33], [42], while later studies found a higher frequency of HIV-1 subtype A1 [39], [64]. The fact that individuals infected with HIV-1 subtype D develop AIDS and die sooner than those infected with subtype A1 [65], [66] could partially explain the decreased prevalence of HIV-1 subtype D in this population. Additionally, it is also possible that HIV-1 subtype D has lower transmission efficiency.

Multiple HIV-1 infections have been reported in previous studies [19], [23], [28], [67]–[69] including in the Mbeya region that reported multiple HIV-1 infections in 19.9% of HIV-1 infected in a high-risk sexual behavior population (female bar and hotel workers) [10]. In this study we identified 12 subjects (27%) with multiple HIV-1 infections including distinct and closely related viral variants. It was not clear whether subjects infected with closely related HIV-1 variants were superinfected. The mean pairwise distances between each subject's clusters were too high for the intra-patient diversity but too low for the inter-patient level of diversity. Due to limitations of the study design, for subjects identified with closely related viral variants, we cannot exclude (1) long history of HIV infection, (2) transmission of multiple viral variants from the same partner with chronic infection (e.g., on multiple occasions), or (3) superinfection with closely related viral variants circulating within the same transmission network. HIV-1 infection with multiple variants dramatically increases the overall diversity and may lead to recombinant viral variants within a short period of time. It is likely that multiple viral variants might contribute to fast exhaustion of immune response and a reduced capacity of the immune system to contain viral replication [70]. HIV-1 infections with multiple variants and recombinants have been associated with faster disease progression [18], [27]–[29] and might lower the likelihood of successful anti-retroviral therapy if multiple infections are caused by both susceptible and drug resistant strains [30].

The epidemiologically linked viruses in subjects 87 and 322 were evident by branching topology and a significant aLRT support. This suggests that both subjects may have been infected from a common source or represent an HIV-1 transmission chain in this community [44], [46]. Similar results were reported previously in five bar and hotel workers who were infected with HIV-1 subtype A1 and clustered together with a significant bootstrap support, suggesting some epidemiological link among the subjects [36].

In this study four of the 50 subjects were infected with inter-subtype recombinant viruses (one case of D/A, two cases of C/A, and one case had two complex mosaic recombinants). The later had one virus comprised of HIV-1 subtype D and unclassified regions that did not cluster with any known HIV-1 group M subtypes. The second virus had an insertion of unclassified region in the background of subtype D. These findings reveal the ongoing recombination of circulating HIV-1 strains which resulted in complex mosaic recombinants.

Viral quasispecies in one subject (322) included HIV-1 subtype A and C/A recombinant providing evidence for a multiple infection. Phylogenetic analyses demonstrated a substantial difference between HIV-1 subtype A sequences and the A fragment in the C/A recombinant, suggesting different origins of non-recombinant and recombinant HIV-1 subtype A in this subject. The available sample did not resolve whether this subject was infected by multiple HIV-1 variants, or contracted a super-infection.

The identified recombinant viruses in this study were unique, and shared no recombination patterns and/or breakpoints suggesting evidence for ongoing recombination among circulating viruses in Tanzania, and emergence of unique recombinant forms (URFs). URFs of HIV-1 with different recombination patterns have been reported previously in Tanzania [10], [16], [36], [38], [71]. No viral sequences closely related to the HIV-1 CRF10_CD reported previously in Tanzania [34], [37], [38] have been found in this study, suggesting no selective advantage of CRF10_CD in this population.

The prevalence of inter-subtype recombinant viruses in this study was lower than in the previous study from Tanzania [36]. To address the observed difference in frequency of inter-subtype recombinants, we applied current analytical methods to HIV-1 sequences from the previous study. Only two of seven sequences reported as recombinants [36] showed evidence for inter-subtype recombination suggesting high sensitivity of recombination analysis to the analytical method used (Table S4). The lower prevalence of inter-subtype recombinants can be explained by at least three reasons. Firstly, the use of SGA/S approach minimizes *in vitro* viral recombination resulting from Taq polymerase-mediated template switching [47], [48]. Secondly, the analysis of only one region of the HIV-1 genome (*env* V1-C5) may underestimate the true proportion of recombinant viruses in this population. Lastly, if some of the recombinants are transient with low levels of replication, then the recombinants could likely go undetected [72]. It has been described that about 10% of the HIV-1 isolates sequenced over large regions including several genes are inter-subtype recombinants [73]. Thus, including multiple regions of the HIV-1 genome or the full HIV-1 genome may provide a better estimate of recombinant viruses [69].

Analysis of the HIV-1 RNA levels in plasma revealed no statistical difference between HIV-1 subtypes in this study. A high HIV-1 plasma viral load was observed in some of the subjects who had HIV-1 multiple infections and recombinant viruses. However, these findings could not be statistically corroborated because of small sample size. Similarly, changes in mean pairwise genetic distances were not statistically significant over the observed period of time, apparently due to high heterogeneity among subjects, relatively small sample size and the short duration of the follow-up period.

This study has limitations. Firstly, the analysis of one region of the HIV-1 genome (*env* V1-C5 of gp 120) may underestimate the true proportion of HIV-1 subtypes, recombinants and multiplicity of HIV-1 infections. Secondly, the duration of HIV-1 infection was unknown. The study had no power to determine whether the multiplicity of the HIV-1 infections were due to co-infections, super-infections or both. Thirdly, some of the subjects had undetectable plasma HIV-1 viral RNA although they tested HIV-1 positive by antibodies tests. It is likely that the undetectable HIV-1 RNA was associated with the low number of the amplified viral quasispecies in some of the subjects, or resulted in negative amplification. The primers mismatch can also attributable in few cases of PCR failure. In addition, we cannot exclude the possibility that some subjects may have been receiving antiretroviral treatment at the time of sample collection without our knowledge.

Therefore, further studies with large sample sizes using multiple regions of the HIV-1 genome or the full HIV-1 genome will be necessary to elaborate the prevalence of multiple HIV-1 infections as well as inter-subtype recombination in this population.

In summary, this study demonstrated that HIV-1 subtype A1 is still the most prevalent subtype followed by HIV-1 subtype C. We also reported for the first time a high prevalence HIV-1 infection with multiple variants in this population in Moshi, Tanzania. Our results suggest that individuals infected with multiple viral variants might contribute to increased HIV-1 diversity through generation of complex recombinant viruses which pose a challenge to the design and development of HIV-1 vaccines, diagnostics and antiretroviral therapy.

## Supporting Information

Figure S1 ML Phylogenetic tree and highlighter plot of subject 530 shows an example of single variant of HIV-1 infection. The legend at the left of the ML tree indicates reference HIV-1 subtypes. The ticks in the highlighter plot represent the following: A: green, T: red, G: yellow, C: light blue: Gaps: gray. Scale at the bottom of the Figure corresponds to 0.1 nucleotide substitutions per site.
(TIF)

Figure S2 Change in pairwise genetic distance (%; means) among subtypes A1, C, D and inter-subtype recombinant viruses over one year of infection.
(TIF)

Table S1 Age distribution, viral load, number of DNA sequences, mean pairwise genetic distances and HIV-1 single/multiple variants.
(DOCX)

Table S2 Comparison of plasma HIV-1 RNA load between HIV-1 subtypes over one year of infection.
(DOCX)

Table S3 Comparison of mean pairwise genetic distance (%) among HIV-1 subtypes A1, C, D and inter-subtype recombinant viruses over one year of infection.
(DOCX)

Table S4 Analysis of HIV-1 proviral DNA sequences (C2–C5 env) with evidence of inter-subtype recombination from a previous study (Kiwelu et al., AIDS Res Hum Retroviruses 2003;19:57–64) by REGA, RIP v. 3.0 and SimPlot.
(DOCX)
