# Supplementary figures and images for: HIV-1 Subtypes and Recombinants in Northern Tanzania: Distribution of Viral Quasispecies
Source: PLoS One. 2012 Oct 31;7(10):e47605. doi: 10.1371/journal.pone.0047605 (PMC3485255; doi:10.1371/journal.pone.0047605)

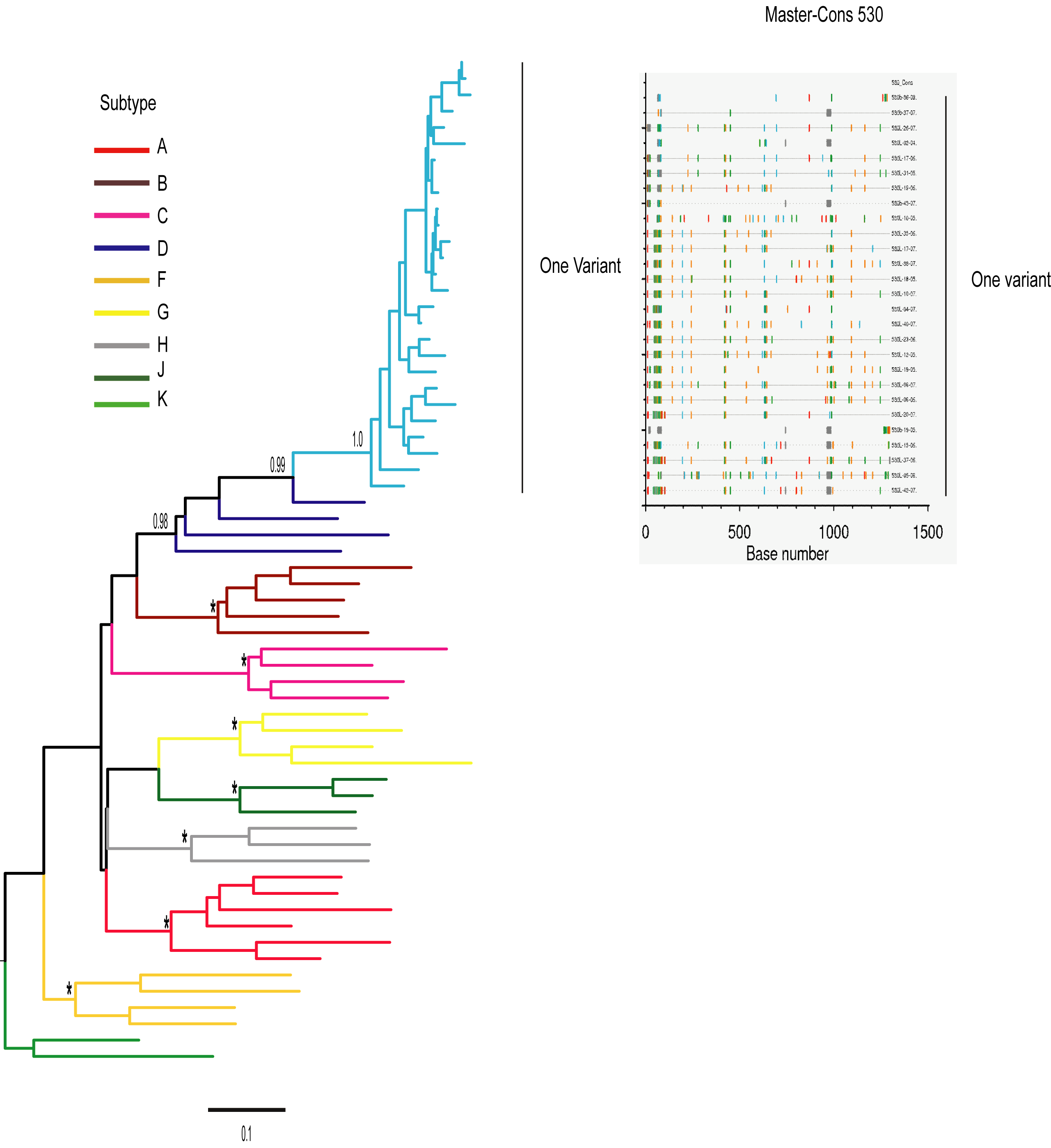

Supplement: Figure S1 — ML Phylogenetic tree and highlighter plot of subject 530 shows an example of single variant of HIV-1 infection. The legend at the left of the ML tree indicates reference HIV-1 subtypes. The ticks in the highlighter plot represent the following: A: green, T: red, G: yellow, C: light blue: Gaps: gray. Scale at the bottom of the Figure corresponds to 0.1 nucleotide substitutions per site. (TIF) [file pone.0047605.s001.tif]

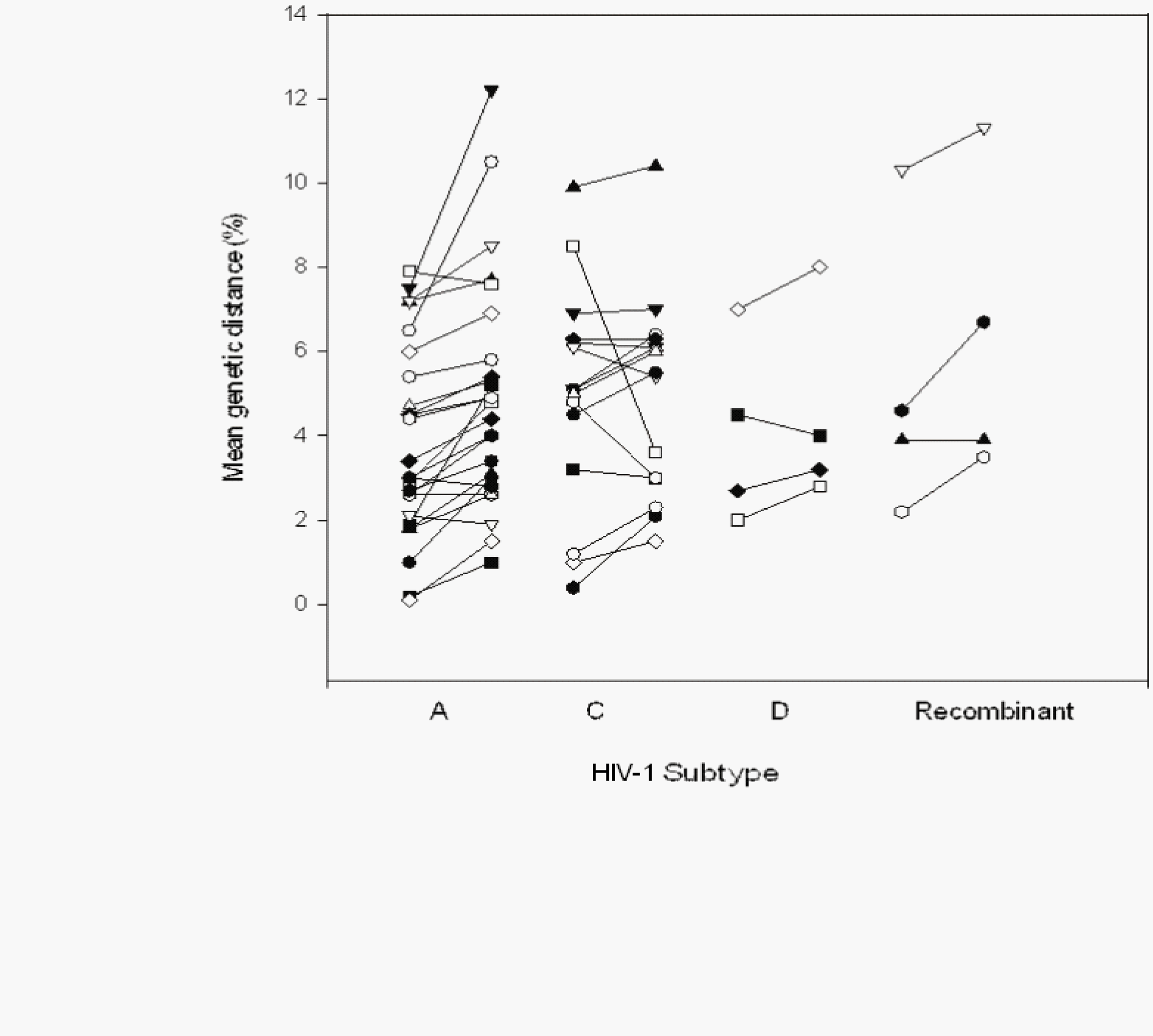

Supplement: Figure S2 — Change in pairwise genetic distance (%; means) among subtypes A1, C, D and inter-subtype recombinant viruses over one year of infection. (TIF) [file pone.0047605.s002.tif]
