# Supplementary material for: HIV-1 Subtypes and Recombinants in Northern Tanzania: Distribution of Viral Quasispecies
Source: PLoS One. 2012 Oct 31;7(10):e47605. doi: 10.1371/journal.pone.0047605 (PMC3485255; doi:10.1371/journal.pone.0047605)
Supplement: Table S1 — Age distribution, viral load, number of DNA sequences, mean pairwise genetic distances and HIV-1 single/multiple variants. (DOCX) [file pone.0047605.s003.docx]

| **Table S1 : Age distribution, viral load, number of DNA sequences, mean pairwise genetic distances and HIV-1 single/multiple**  **variants** | | | | | | | | | |
| --- | --- | --- | --- | --- | --- | --- | --- | --- | --- |
| Subject code | Age | HIV-1Subtype | Plasma HIV-1 RNA load Log_10_ copies/ml | Plasma HIV-1 RNA load Log_10_ copies/ml | Number of *env* DNA sequences | | Mean pairwise genetic distance (%) | | HIV-1 viral variant |
|  |  |  | Viral load baseline visit | Viral load  12 month visit | Baseline visit DNA/RNA* | 12 month visit DNA/RNA* | Baseline visit DNA/RNA* | 12 month visit DNA |  |
| 7 | 50 | A1 | 3.7 | 3.9 | 14 | 4 | 1.0 | 2.4 | single |
| 86 | 30 | A1 | 4.2 | 3.9 | 2 | 3 | 1.9 | 5.2 | single |
| 107 | 34 | A1 | 5.3 | 2.3 | 13 | 20 | 5.3 | 5.4 | single |
| 177 | 28 | A1 | 4.9 | 3.7 | 36 | 26 | 7.2 | 7.7 | single |
| 178 | 53 | A1 | 4.0 | 4.3 | 7 | 17 | 2.1 | 1.9 | single |
| 181 | 33 | A1 | 3.7 | 3.8 | N/A | 1 | N/A | N/A | N/A |
| 190 | 40 | A1 | 2.3 | 2.3 | 7 | 9 | 2.6 | 4.0 | single |
| 204 | 38 | A1 | 3.2 | 2.3 | 21 | 20 | 2.6 | 2.6 | single |
| 209 | 22 | A1 | 4.1 | 4.5 | 15 | 22 | 2.7 | 3.4 | single |
| 355 | 25 | A1 | 4.2 | 4.9 | 12 | 29 | 1.8 | 2.6 | single |
| 404 | 29 | A1 | 3.2 | 3.6 | N/A | 30 | N/A | 2.9 | single |
| 405 | 29 | A1 | 3.2 | 4.4 | N/A | 17 | N/A | 2.6 | single |
| 491 | 37 | A1 | 4.6 | 4.5 | 48/14* | 39 | 0.1* | 1.3 | single |
| 697 | 30 | A1 | 4.0 | 4.2 | 5 | 27 | 3.4 | 4.4 | single |
| 794 | 23 | A1 | 4.8 | 5.3 | 19 | 22 | 0.1 | 1.5 | single |
| 807 | 22 | A1 | 4.4 | 4.8 | 22 | 19 | 1.8 | 3.1 | single |
| 905 | 45 | A1 | 4.5 | 4.4 | 15 | 13 | 7.2 | 8.6 | single |
| 909 | 26 | A1 | 5.7 | 4.9 | 18/13* | 42 | 4.5* | 4.9 | single |
| 945 | 31 | A1 | 4.4 | 4.9 | 16 | 19 | 3.0 | 2.8 | single |
| 20 | 21 | A1 | 5.1 | 5.0 | 15/30* | 31 | 6.6* | 11.0 | multiple |
| 46 | 21 | A1 | 5.1 | 4.8 | 13 | 15 | 4.5 | 5.3 | multiple |
| 65 | 32 | A1 | 4.9 | 5.3 | 24/11* | 28 | 4.8 | 5.5 | multiple |
| 87 | 29 | A1 | 4.3 | 5.0 | 16 | 21 | 2.9 | 4.8 | multiple |
| 168 | 30 | A1 | 5.6 | 5.5 | 14/25* | 16 | 5.7* | 6.3 | multiple |
| 237 | 40 | A1 | 5.5 | 4.7 | 29 | 11 | 5.2 | 5.8 | multiple |
| 245 | 29 | A1 | 5.4 | 2.3 | 22/13* | 15 | 7.0* | 8.8 | multiple |
| 620 | 35 | A1 | 5.1 | 5.2 | 16 | 22 | 7.5 | 5.6 | multiple |
| 740 | 30 | A1 | 6.3 | 2.3 | 18 | N/A | 5.6 | N/A | multiple |
| 27 | 41 | C | 6.0 | 2.3 | 34/19* | 5 | 5.1* | 6.4 | single |
| 63 | 35 | C | 5.3 | 2.3 | 5 | 5 | 5.1 | 5.8 | single |
| 66 | 36 | C | 4.2 | 4.5 | 5 | 27 | 7.0 | 5.6 | single |
| 80 | 28 | C | 3.2 | 3.6 | 9 | 23 | 2.6 | 3.5 | single |
| 276 | 22 | C | 4.5 | 4.5 | 16/13* | 27 | 1.0* | 1.5 | single |
| 321 | 27 | C | 4.3 | 4.0 | N/A | 28 | N/A | 3.9 | single |
| 497 | 22 | C | 4.8 | 4.9 | 25 | 6 | 0.4 | 2.1 | single |
| 498 | 28 | C | 4.9 | 2.3 | 3 | 11 | 4.8 | 3.0 | single |
| 558 | 35 | C | 5.5 | 2.8 | 18/16* | 13 | 5.9* | 5.5 | single |
| 603 | 46 | C | 5.0 | 3.7 | 19 | 21 | 4.6 | 5.7 | single |
| 838 | 26 | C | 4.2 | 4.5 | 17 | 28 | 1.2 | 2.1 | single |
| 968 | 38 | C | 5.4 | 5.2 | 24 | 24 | 6.9 | 7.0 | single |
| 171 | 38 | C | 5.0 | 2.3 | 21 | 4 | 8.5 | 3.6 | multiple |
| 201 | 24 | C | 2.7 | 2.3 | 26 | 12 | 7.6 | 7.4 | multiple |
| 291 | 22 | C | 5.1 | 5.5 | 16/22* | 25 | 10.1* | 10.5 | multiple |
| 530 | 23 | D | 4.0 | 4.0 | 4 | 23 | 2.9 | 4.0 | single |
| 733 | 37 | D | 5.3 | 5.2 | 29/19* | 23/24* | 2.0* | 2.9 | single |
| 871 | 32 | D | 2.5 | 3.2 | 3 | 10 | 2.6 | 3.3 | single |
| 33 | 30 | D/A | 3.5 | 3.4 | 21/1* | 26 | 3.9* | 3.9 | N/D |
| 322 | 31 | A1, C/A | 4.1 | 4.2 | 16/9* | 16 | 10.3* | 11.3 | N/D |
| 471 | 47 | C/A | 5.6 | 3.3 | 29 | 21 | 2.2 | 3.5 | N/D |
| 510 | 23 | D/U^§^, D/U^§^/D | 4.3 | 4.4 | 22/10* | 25 | 4.6* | 6.7 | N/D |

RNA*-Amplified from RNA, U^§^- Unclassified region, N/A – Not available
