# Supplementary material for: HIV-1 Subtypes and Recombinants in Northern Tanzania: Distribution of Viral Quasispecies
Source: PLoS One. 2012 Oct 31;7(10):e47605. doi: 10.1371/journal.pone.0047605 (PMC3485255; doi:10.1371/journal.pone.0047605)
Supplement: Table S2 — Comparison of plasma HIV-1 RNA load between HIV-1 subtypes over one year of infection. (DOCX) [file pone.0047605.s004.docx]

**Table S2**

Comparison of plasma HIV-1 RNA load between HIV-1 subtypes over one year of infection.

| HIV-1 subtype | Baseline visit | 12 month visit |
| --- | --- | --- |
|  | P-value | |
| A vs. C | 0.491 | 0.131 |
| A vs. D | 0.340 | 0.942 |
| A vs. Recombinant | 0.827 | 0.506 |
| C vs. D | 0.236 | 0.517 |
| C vs. Recombinant | 0.550 | 0.960 |
| D vs. Recombinant | 0.628 | 1.000 |
