# Supplementary material for: HIV-1 Subtypes and Recombinants in Northern Tanzania: Distribution of Viral Quasispecies
Source: PLoS One. 2012 Oct 31;7(10):e47605. doi: 10.1371/journal.pone.0047605 (PMC3485255; doi:10.1371/journal.pone.0047605)
Supplement: Table S3 — Comparison of mean pairwise genetic distance (%) among HIV-1 subtypes A1, C, D and inter-subtype recombinant viruses over one year of infection. (DOCX) [file pone.0047605.s005.docx]

**Table S3**

Comparison of mean pairwise genetic distance (%) among HIV-1 subtypes A1, C, D and inter-subtype recombinant viruses over one year of infection.

|  | Overall mean pairwise genetic distance (%) | |  |
| --- | --- | --- | --- |
| HIV-1 subtype | Baseline visit | 12 month visit | P-value |
| A1 | 3.884 ± 2.265 | 4.631 ± 2.423 | 0.262 |
| C | 5.057 ± 2.922 | 4.907 ± 2.421 | 0.881 |
| D | 2.500 ± 0.458 | 3.400 ± 0.557 | 0.097 |
| Recombinants | 5.250 ± 3.514 | 6.350 ± 3.594 | 0.677 |
