# Supplementary material for: HIV-1 Subtypes and Recombinants in Northern Tanzania: Distribution of Viral Quasispecies
Source: PLoS One. 2012 Oct 31;7(10):e47605. doi: 10.1371/journal.pone.0047605 (PMC3485255; doi:10.1371/journal.pone.0047605)
Supplement: Table S4 — Analysis of HIV-1 proviral DNA sequences (C2–C5 env) with evidence of inter-subtype recombination from a previous study (Kiwelu et al., AIDS Res Hum Retroviruses 2003;19:57–64) by REGA, RIP v. 3.0 and SimPlot. (DOCX) [file pone.0047605.s006.docx]

**Table S4**

Analysis of HIV-1 proviral DNA sequences (C2-C5 env) with evidence of inter-subtype recombination from the previous study (Kiwelu et al., 2003) by REGA, RIP v. 3.0 and SimPlot.

| IDNO |  | Current methods used and HIV-1 subtypes | | |
| --- | --- | --- | --- | --- |
|  | Previous results | REGA | SimPlot | RIP v. 3.0 |
| TZM047019 | A/D | D | D | D |
| TZM047115 | A/D | D | D | D |
| TZM047121 | A/D | N/A | D/A | A1/D/A1 |
| TZM047132 | A2/C | N/A | C/A1 | A1/C/A1 |
| TZM047267 | A/C | N/A | A1 | A1 |
| TZM047308 | A/C/D | N/A | U^§^/A1 | A1 |
| TZM047390 | A/C | N/A | A1 | A1 |

N/A: Not available

^§^ Unclassified region
